# Oral microbiome and nitric oxide biomarkers in older people with mild cognitive impairment and *APOE4* genotype

**DOI:** 10.1093/pnasnexus/pgae543

**Published:** 2025-01-28

**Authors:** Joanna E L’Heureux, Anne Corbett, Clive Ballard, David Vauzour, Byron Creese, Paul G Winyard, Andrew M Jones, Anni Vanhatalo

**Affiliations:** Faculty of Health and Life Sciences, University of Exeter Medical School, University of Exeter, St Luke's campus, Exeter EX1 2LU, United Kingdom; Faculty of Health and Life Sciences, University of Exeter Medical School, University of Exeter, St Luke's campus, Exeter EX1 2LU, United Kingdom; Faculty of Health and Life Sciences, University of Exeter Medical School, University of Exeter, St Luke's campus, Exeter EX1 2LU, United Kingdom; Norwich Medical School, University of East Anglia, Norwich NR4 7TJ, United Kingdom; Department of Life Sciences, University of Brunel, London UB8 3PH, United Kingdom; Faculty of Health and Life Sciences, University of Exeter Medical School, University of Exeter, St Luke's campus, Exeter EX1 2LU, United Kingdom; Faculty of Health and Life Sciences, University of Exeter Medical School, University of Exeter, St Luke's campus, Exeter EX1 2LU, United Kingdom; Faculty of Health and Life Sciences, University of Exeter Medical School, University of Exeter, St Luke's campus, Exeter EX1 2LU, United Kingdom

**Keywords:** cognitive status, aging, nitrate, *Prevotella*, genetic risk

## Abstract

Apolipoprotein *E4* (*APOE4*) genotype and nitric oxide (NO) deficiency are risk factors for age-associated cognitive decline. The oral microbiome plays a critical role in maintaining NO bioavailability during aging. The aim of this study was to assess interactions between the oral microbiome, NO biomarkers, and cognitive function in 60 participants with mild cognitive impairment (MCI) and 60 healthy controls using weighted gene co-occurrence network analysis and to compare the oral microbiomes between *APOE4* carriers and noncarriers in a subgroup of 35 MCI participants. Within the MCI group, a high relative abundance of *Neisseria* was associated with better indices of cognition relating to executive function (Switching Stroop, *r_s_* = 0.33, *P* = 0.03) and visual attention (Trail Making, *r_s_* = −0.30, *P* = 0.05), and in the healthy group, *Neisseria* correlated with working memory (Digit Span, *r_s_* = 0.26, *P* = 0.04). High abundances of *Haemophilus* (*r_s_* = 0.38, *P* = 0.01) and *Haemophilus parainfluenzae* (*r_s_* = 0.32, *P* = 0.03), that co-occurred with *Neisseria* correlated with better scores on executive function (Switching Stroop) in the MCI group. There were no differences in oral nitrate (*P* = 0.48) or nitrite concentrations (*P* = 0.84) between the MCI and healthy groups. Linear discriminant analysis Effect Size identified *Porphyromonas* as a predictor for MCI and *Prevotella intermedia* as a predictor of *APOE4*-carrier status. The principal findings of this study were that a greater prevalence of oral *P. intermedia* is linked to elevated genetic risk for dementia (*APOE4* genotype) in individuals with MCI prior to dementia diagnosis and that interventions that promote the oral *Neisseria–Haemophilus* and suppress *Prevotella*-dominated modules have potential for delaying cognitive decline.

**Significance Statement**

Cognition typically declines during aging and mild cognitive impairment (MCI) may progress to the development of Alzheimer's disease (AD). Periodontal disease-causing bacteria have been linked to worsened cognitive function during aging and the development of AD, which may involve dysfunction of the nitrate–nitrite–nitric oxide pathway. We found that the oral *Porphyromonas* genus was associated with MCI and that the abundance of *Prevotella intermedia* was a predictor of apolipoprotein *E4*–carrier status. The balance between two metabolic pathways for oral nitrate reduction, denitrification, and dissimilatory nitrate reduction to ammonia (DNRA) was skewed toward DNRA in MCI. These findings have significant implications for understanding preclinical cognitive risk states and how cognitive decline could be delayed or prevented using prebiotic interventions.

## Introduction

Cognitive decline is typically associated with aging (1). In some individuals, clinically significant decline leads to the development of mild cognitive impairment (MCI), which affects about 15% of older adults. MCI is the greatest risk factor for the development of dementia or Alzheimer's disease (Ad), with an estimated 10% of people with MCI converting to dementia each year (2). Cognitive decline and dementia represent a major public health issue, and there is an urgent need to elucidate the risk factors for decline and explore means to reduce this risk (3).

Periodontitis has been associated with worsened cognitive function (4) and missing teeth in the oral cavity have been associated with lower Mini Mental State Examination test scores (5). A potential mechanism linking oral health and cognitive decline is the increased inflammation and damage caused by pathogenic oral bacteria, such as *Porphyromonas gingivalis*, *Treponema denticola*, and *Prevotella intermedia*, which in turn increase the risk for periodontal diseases (6). Patients with Ad present with higher levels of *P. gingivalis* and lower bacterial diversity in the oral cavity compared with healthy controls (7, 8). Two routes have been suggested by which oral pathogens may cause cognitive decline. The direct route is via trauma in the mouth, whereby the oral bacteria gain access to the circulatory system and then relocate to the brain by traversing the blood–brain barrier (9), which is increasingly permeable in Ad. Known oral disease-causing bacteria have been found in the cerebrospinal fluid of patients with a brain abscess (10), and *P. gingivalis* has been discovered in the brains of patients with Ad (11). The oral bacteria may also indirectly affect the brain by impairing the oral mucosal barrier and allowing metabolites produced by the oral bacteria to enter the circulatory system or exacerbating inflammation through the overproduction of cytokines (12, 13).

One potential mechanism linking oral health and cognition is the production of nitric oxide (NO) via the nitrate–nitrite–NO pathway (14). NO is an important signaling molecule for many physiological processes, such as vasodilation, muscle contraction, neurotransmission, and host defense against microorganisms (15–17). There are two known methods of NO production in mammals. The L-arginine pathway endogenously produces NO via NO synthase (NOS) enzymes (18). The second mechanism is through the nitrate–nitrite–NO pathway, where commensal oral bacteria reduce nitrate to nitrite, which is further reduced to form NO in the circulation and in tissues (19). A main role of NO in the brain is binding to guanylyl cyclase which acts as a pre- or postsynaptic messenger. In preclinical models, NOS activity has been detected in the hippocampus, which is the brain region responsible for learning and memory, and inhibition of NOS resulted in impaired memory (20). NO has a role in synaptic plasticity and long-term potentiation, and a reduction in NO availability may be linked with the inability of patients with Ad to retain new information (21, 22). During aging, endogenous NO production is reduced through decreased NOS gene expression and increased degradation of arginine, catalyzed by arginase, which is associated with cardiovascular diseases such as hypertension and risk of vascular Ad (23–25). Decreased NO biomarker concentrations of nitrate and nitrite in the plasma and brains of patients with Ad have been reported compared with healthy controls (26–28).

A key genetic risk factor for cognitive decline and Ad is the apolipoprotein *E4* (*APOE4*) allele, which has been associated with weakening of the blood–brain barrier (29). *APOE4* carriers also have an elevated risk for other systemic diseases, such as hypertension, atherosclerosis, and skeletal muscle weakness (30–33), and these conditions are also notably hallmarked by NO deficiency. This suggests that there may be interactions between *APOE4*, NO bioavailability and the oral microbiome during aging-associated cognitive decline. It is, however, unknown whether characteristics of the oral microbiome correlate with cognitive function, or whether changes in oral microbiome composition may already be detected in healthy older individuals with MCI but prior to dementia diagnosis.

The purpose of this study was, therefore, to compare the oral microbiomes and oral NO biomarkers between individuals with MCI and healthy controls, and explore relationships between co-occurring modules of oral bacteria, cognitive function, and NO biomarkers. A secondary aim was to perform an MCI subgroup analysis to compare oral microbiomes and NO biomarkers between *APOE4* carriers and noncarriers.

## Results

### Participant characteristics

There were 120 participants recruited in the study. After data preprocessing, 5 samples were removed from further analysis due to low-quality data. Therefore, 115 samples were analyzed in total. Included in the data analysis were 60 participants in the healthy group (17 males; mean age ± SD, 67 ± 5 years; 43 females; mean age ± SD, 67 ± 8 years) and 55 participants in the MCI group (8 males; mean age ± SD, 70 ± 6 years; 47 females; mean age ± SD, 68 ± 8 years). *APOE* status was defined as “high-risk” *APOE4* carriers (*E3E4*, *E4E4*) and “low-risk” *APOE4* noncarriers (*E2E3*, *E3E3*) in a subset of MCI participants. *E2E4* carriers (*n* = 2) were excluded from this analysis due to ambiguous risk status, and therefore, 33 participants were included in the *APOE4* subgroup analysis (Table 1).

**Table 1.** Cohort *APOE4* status characteristics.

Characteristic	*APOE4* carrier (*n* = 14)	*APOE4* noncarrier (*n* = 19)
Risk group	High	Low
*APOE* status (*n*)	*E3E4* (*n* = 13) *E4E4* (*n* = 1)	*E2E3* (*n* = 4) *E3E3* (*n* = 15)
Male sex (*n*)	3	3
Female sex (*n*)	11	16
Male age (mean years ± SD)	68 ± 6	70 ± 7
Female age (mean years ± SD)	68 ± 7	69 ± 8

### Mouth rinse nitrate and nitrite concentrations

**Fig. 1.**
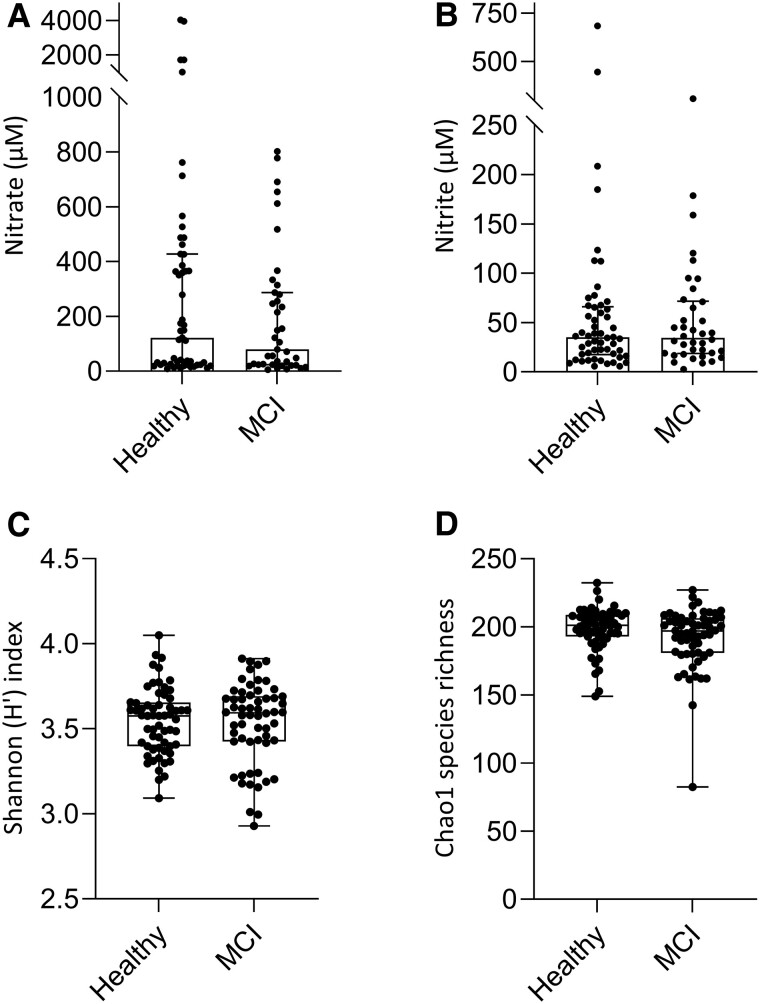
Mouth rinse nitrate and nitrite concentrations and species diversity. The median mouth rinse nitrate (A) and nitrite (B) concentrations were not different between healthy and MCI groups. There were also no differences in median Shannon H′ index (C) or Chao1 species richness (D) between the healthy and MCI groups. Error bars in (A) and (B) indicate IQR. The box plots in (C) and (D) represent the median and IQR, while whiskers show the minimum and maximum values.

There were no differences in mouth rinse nitrate (median ± interquartile range [IQR]; healthy 122 ± 403 µM; MCI 80 ± 263 µM, *P* = 0.48) or nitrite concentrations (median ± IQR; healthy 35 ± 48 µM; MCI 34 ± 53 µM, *P* = 0.84) between the healthy and MCI groups (Fig. 1A and B). Within the MCI group, there were no differences between *APOE4* carriers (118 ± 110 µM) and noncarriers (206 ± 258 µM) in mouth rinse nitrate (*P* = 0.30) or nitrite concentrations (carriers 42 ± 46 µM, noncarriers 74 ± 48 µM, *P* = 0.12).

### Alpha and beta diversity of the oral microbiome

Species diversity was assessed using the Shannon H′ index, and species richness was measured using Chao1. The Mann–Whitney *U* test revealed that there were no differences in Shannon H′ index (*P* = 0.75, Fig. 1C) or Chao1 species richness (*P* = 0.45, Fig. 1D) between the healthy and MCI groups. There were also no differences in Shannon H′ diversity (*P* = 0.32) or in Chao1 species richness (*P* = 0.92) between *APOE4* carriers and noncarriers.

**Fig. 2.**
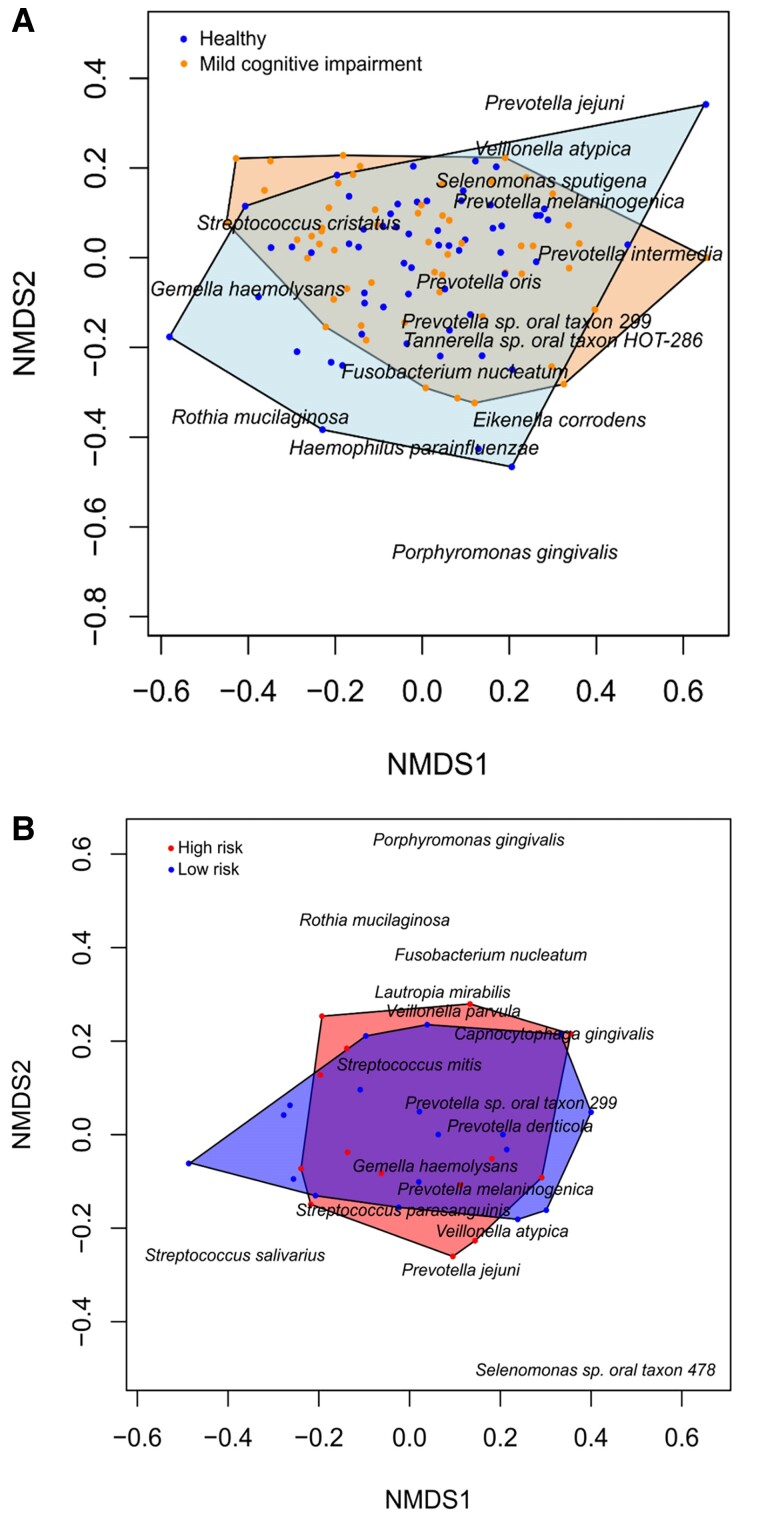
NMDS of healthy and MCI groups. (A) NMDS showed no distinct groupings or significant differences in the oral microbiomes between healthy (blue) and MCI groups (orange; *P* = 0.80). B) NMDS also indicated no distinct groupings or significant differences between the *APOE4* carriers (red) and noncarriers (blue; *P* = 0.30) within the MCI group. NMDS was based on Bray–Curtis dissimilarity, and the data points represent the oral microbiome of each individual participant. Only selected species of highly abundant species are shown in the plot for visual clarity.

Nonmetric multidimensional scaling (NMDS) was used to explore possible underlying patterns in the microbiome data. Stress was 0.01, meaning that the individual distances between the objects were well represented. The NMDS showed that the oral microbiomes of healthy and MCI participants displayed no distinct groupings and that there were no significant differences between the healthy and MCI groups (*P* = 0.80, Fig. 2A) or between *APOE4* carriers and noncarriers (*P* = 0.70, Fig. 2B).

### Weighted gene co-occurrence network analysis of the oral microbiome and cognitive test outcomes

**Fig. 3.**
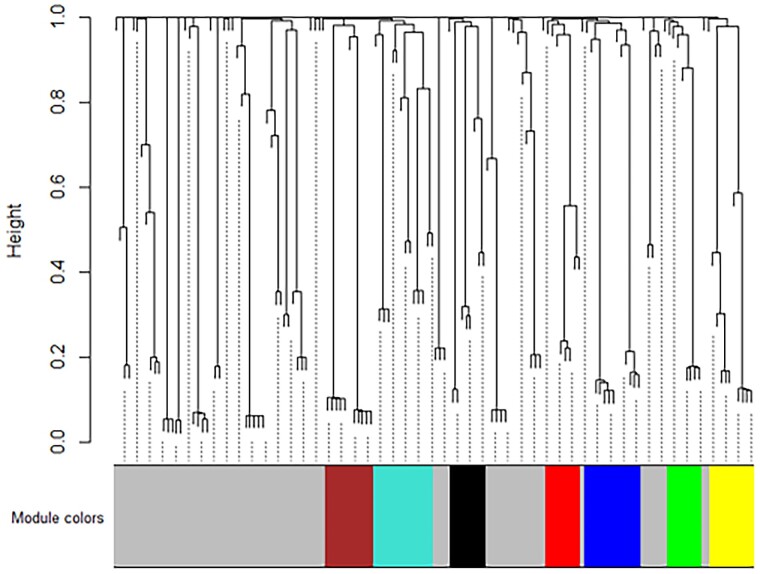
Consensus gene dendrogram, generated using a signed network where modules represent positively correlated taxonomic units. Seven distinct microbiome modules (ME1–ME7) were identified.

Due to the similarities in bacterial diversity between the healthy and MCI groups, a consensus correlation network was used to assess consistencies among the results of healthy and MCI groups. The weighted gene co-occurrence network analysis (WGCNA) analysis revealed seven consensus modules of oral bacteria, which were given arbitrary colors and labeled from ME0 to ME7 (Fig. 3). ME0 contained the operational taxonomic units (OTUs) which did not significantly correlate with other OTUs and were thus not assigned to a module. Table 2 provides a list of the species and genera assigned to modules ME1–ME7.

**Table 2.** OTUs assigned to consensus WGCNA modules.

Module	Module color	OTU
ME1	Black	|g__*Lachnoanaerobaculum*
		|g__*Mogibacterium*
		|s__*Lachnoanaerobaculum umeaense*
		|s__*Mogibacterium diversum*
ME2	Blue	|g *Lancefieldella*
		|g__*Megasphaera*
		|g__*Veillonella*
		|s__*Actinomyces pacaensis*
		|s *Lancefieldella parvula*
		|s__*Veillonella atypica*
		|s__*Veillonella parvula*
ME3	Red	|g__*Prevotella*
		|s__*P. intermedia*
		|s__*P. jejuni*
		|s__*P. melaninogenica*
ME4	Turquoise	|g__*Fusobacterium*
		|g__*Leptotrichia*
		|g__*Porphyromonas*
		|g__*Tannerella*
		|s__*Fusobacterium nucleatum*
		|s__*Porphyromonas gingivalis*
		|s__*Tannerella* sp. oral taxon HOT-286
ME5	Brown	|g__*Dialister*
		|g__*Parvimonas*
		|g__*Treponema*
		|s__*Parvimonas micra*
ME6	Yellow	|g__*Haemophilus*
		|g__*Neisseria*
		|s__*Haemophilus parainfluenzae*
ME7	Green	|g__*Capnocytophaga*
		|s__*Campylobacter showae*
		|s__*Capnocytophaga gingivalis*
		|s__*Capnocytophaga sputigena*

Only the assigned species and genus are shown for brevity.

**Fig. 4.**
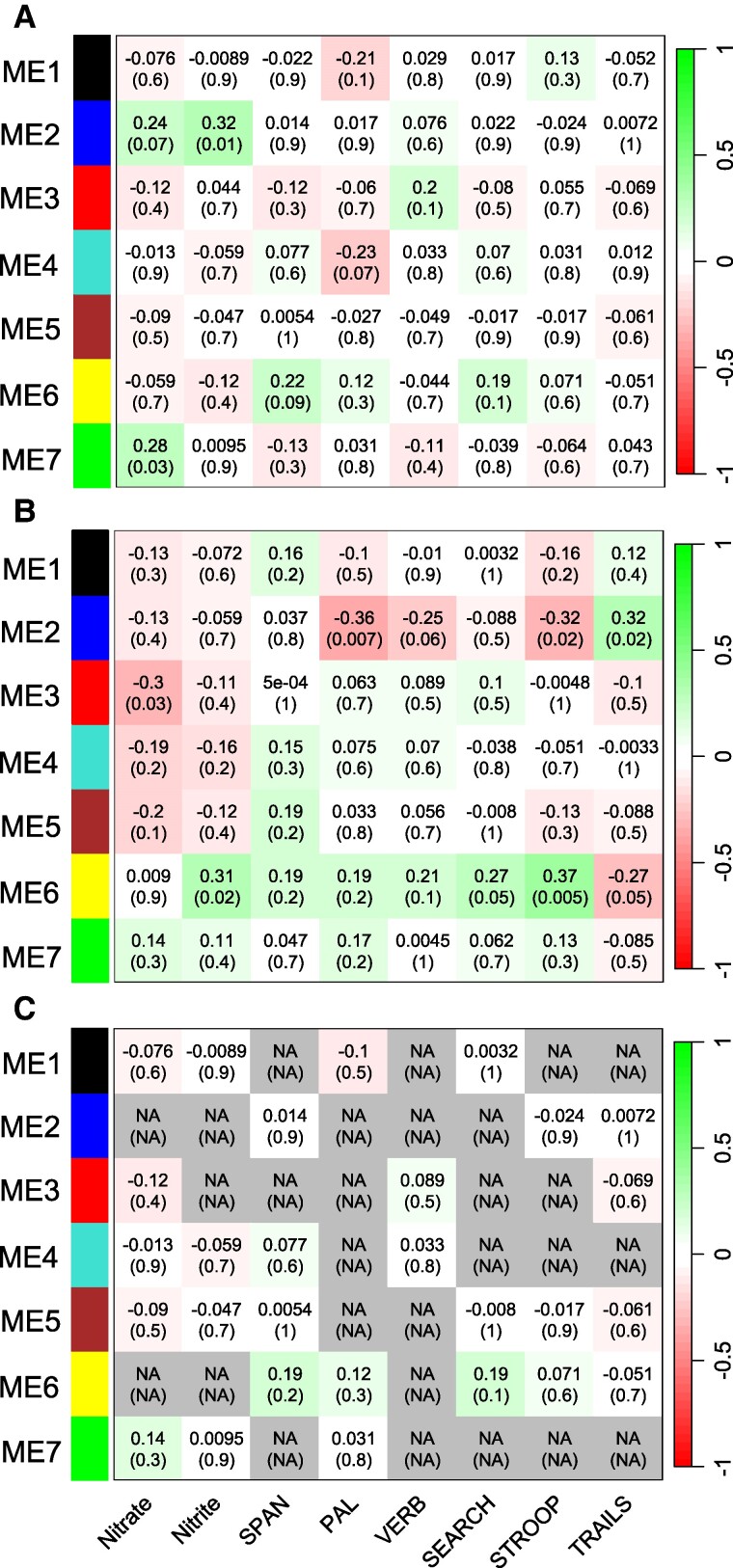
Signed WGCNA heat maps for the healthy (A) and MCI (B) groups, together with a consensus network (C) combining the two groups. Positive correlations between modules and traits are shown in green, whereas negative correlations are shown in red. The first cell value denotes the *r*-value and the second value (in parentheses) denotes the *P*-value. The cognitive tests included Digit Span (SPAN), Paired Associates Learning (PAL), Verbal Reasoning (VERB), Self-ordered Search (SEARCH), Switching Stroop (STROOP), and Trail Making (TRAILS).

The cognitive test outcomes included in the network analysis included measures of working memory (Digit Span, Paired Associates Learning, Self-ordered Search), executive function (Verbal Reasoning, Switching Stroop), and visual attention (Trail Making). High scores in tasks on working memory and executive function indicate intact cognitive function, whereas high scores in the Trail Making test (which includes a time dimension) indicate impaired cognitive function in the visual attention domain. The network correlations were notably distinct between the healthy and MCI groups for microbiome modules ME2 (dominated by *Veillonella* and *Megasphaera*), ME3 (dominated by *Prevotella*), and ME6 (dominated by *Neisseria* and *Haemophilus*; Fig. 4A and B). A consensus network indicated that there were no significantly conserved correlations when the groups were combined (Fig. 4C). In the healthy group, ME2 correlated positively with oral nitrite and ME7 (dominated by *Capnocytophaga*) with oral nitrate concentration, while there were no significant correlations between any of the microbiome modules and the cognitive function tests (Fig. 4A). In the MCI group, the ME2 module did not correlate with NO biomarkers, but this module correlated negatively with working memory (Paired Associates Learning task; *r_s_* = −0.36, *P* = 0.007) and executive function (Switching Stroop task; *r_s_* = −0.32, *P* = 0.02), and positively with a Trail Making task of visual attention, where higher scores are indicative of cognitive decline (*r_s_* = 0.32, *P* = 0.02; Fig. 4B). In the MCI group, the ME6 module (dominated by *Neisseria* and *Haemophilus*) correlated positively with oral nitrite concentration (*r_s_* = 0.31, *P* = 0.02), cognitive test outcomes on working memory (Self-Ordered Search task; *r_s_* = 0.27, *P* = 0.05), and executive function (Switching Stroop task; *r_s_* = 0.37, *P* = 0.005; Fig. 4B), and correlated negatively with the Trail Making score (high Trail Making score indicating impaired visual attention; *r_s_* = −0.27, *P* = 0.05; Fig. 4B).

**Fig. 5.**
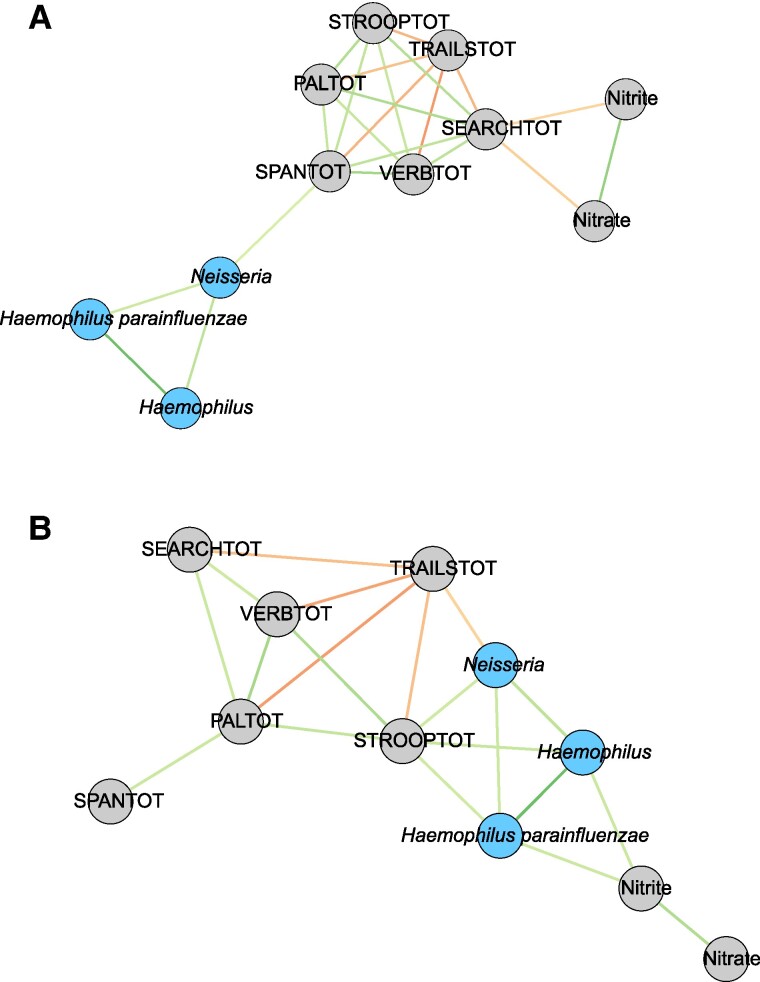
Correlation networks in the healthy (A) and MCI (B) groups show the significantly correlated clinical traits (gray), together with *Haemophilus* and *Neisseria* from ME6 (blue). The line color denotes the direction of the correlation where green is positive, and red is negative. The color brightness indicates the strength of the correlation. The cognitive variables included total scores (TOT) in Digit Span (SPAN), Paired Associates Learning (PAL), Verbal Reasoning (VERB), Self-ordered Search (SEARCH), Switching Stroop (STROOP), and Trail Making (TRAILS) tasks.

The ME6 module, with positive interactions with cognition in the MCI group, was explored further by creating genus- and species-level Spearman correlation networks with cognitive outcomes and NO biomarkers (Fig. 5). In the healthy group, *Neisseria* correlated with Digit Span summary score which assesses working memory (*r_s_* = 0.26, *P* = 0.04; Fig. 5A). In the MCI group, *Neisseria* (*r_s_* = 0.33, *P* = 0.03), *Haemophilus* (*r_s_* = 0.38, *P* = 0.01), and *Haemophilus parainfluenzae* (*r_s_* = 0.32, *P* = 0.03) correlated with Switching Stroop summary score which assesses executive function (Fig. 5B), and *Haemophilus* (*r_s_* = 0.35, *P* = 0.03) and *H. parainfluenzae* (*r_s_* = 0.34, *P* = 0.04) correlated with nitrite concentration (Fig. 5B). In the MCI group, *Neisseria* also negatively correlated with the Trail Making task score, where high scores are indicative of impaired visual attention (*r_s_* = −0.30, *P* = 0.05).

### Linear discriminant analysis effect size comparisons between *APOE4* carriers and noncarriers

**Fig. 6.**
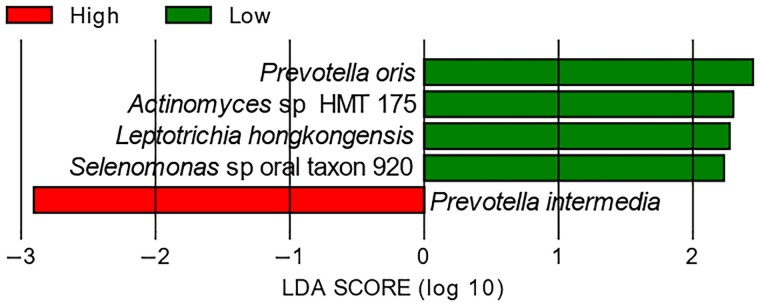
LEfSe showed five significantly abundant taxonomic units differed by *APOE4* status. *APOE4* carriers were defined as *E3E4*/*E4E4* and noncarriers as *E2E*3/*E3E3* allele carriers. Negative bars indicate that the taxonomic unit was significantly abundant in the *APOE4* carriers, and positive bars indicate greater abundance in the noncarriers.

Linear discriminant analysis effect size (LEfSe) analysis identified *Porphyromonas* as a potential biomarker in the MCI group compared with healthy controls (log-linear discriminant analysis [log LDA] score, 2.34; *P* = 0.03). *APOE4* risk status was available for a subset of the participants (Table 1), where LEfSe showed that in *APOE4* carriers, *P. intermedia* was more abundant (log LDA score, 2.96, *P* = 0.04; Fig. 6), and *Leptotrichia hongkongensis*, *Selenomonas* sp. oral taxon 920, *Actinomyces* sp. HMT 175, and *Prevotella oris* were less abundant compared with the *APOE4* noncarriers (Fig. 6).

**Fig. 7.**
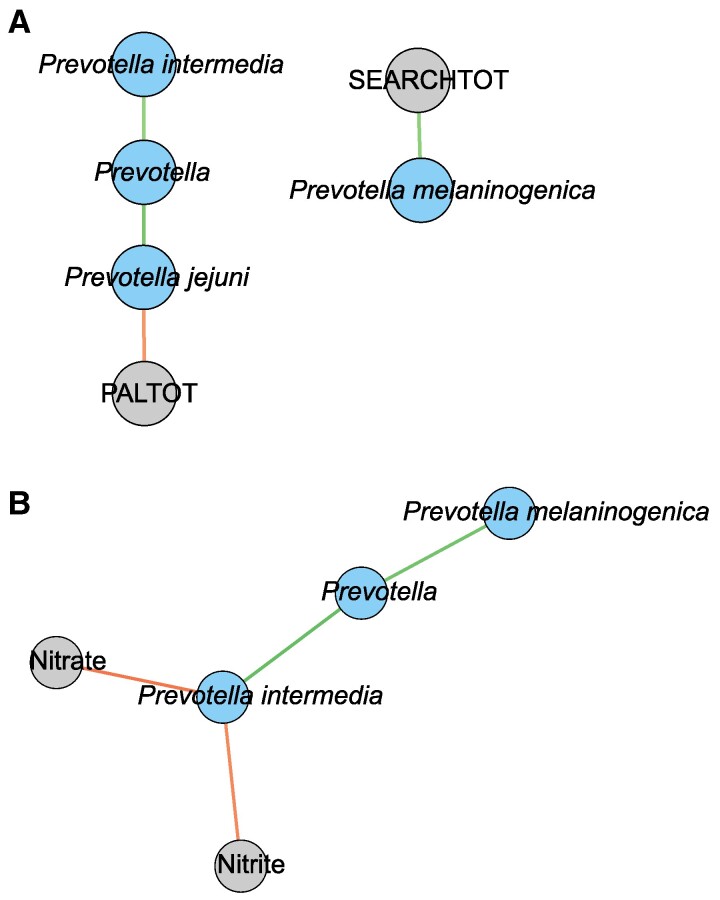
Correlation networks in *APOE4* carriers (A) and noncarriers (B) of the MCI group showing the significantly correlated clinical traits (gray) and OTUs (blue) from the microbiome module ME3 where *Prevotella* was a dominant genus. The line color denotes the direction of the correlation where green is positive, and red is negative. The color brightness indicates the strength of the correlation. The cognitive variables include total scores (TOT) in Paired Associates Learning (PAL) and Self-ordered Search (SEARCH).

To further explore the relationships between *Prevotella*, NO biomarkers, cognitive performance, and *APOE* status, we generated a Spearman correlation network using the WGCNA ME3 module (Fig. 7). In the *APOE4* carrier group, *Prevotella melaninogenica* correlated positively with working memory assessed by Self-ordered Search task (*r_s_* = 0.58, *P* = 0.03), while *Prevotella jejuni* correlated negatively with the Paired Associates Learning task which also assesses working memory (*r_s_* = −0.56, *P* = 0.04, Fig. 7A). In the *APOE4* noncarrier group, mouth rinse nitrate (*r_s_* = −0.73, *P* = 0.007) and nitrite concentrations (*r_s_* = −0.64, *P* = 0.04) correlated negatively with *P. intermedia* (Fig. 7B).

## Discussion

This study characterized oral microbiome modules that are associated with cognitive status, NO homeostasis, and genetic risk for dementia in the Platform for Research Online to investigate Genetics and Cognition in Ageing (PROTECT) cohort of people over 50 years of age who have not received a dementia diagnosis. The principal original findings were that in people with MCI, a high relative abundance of *Neisseria–Haemophilus* module of co-occurring oral bacteria (ME6) was associated with better cognitive outcomes on working memory, executive function, and visual attention, as well as a greater oral nitrite concentration, while a high abundance of the oral *Prevotella*-dominated module (ME3) was associated with low oral nitrate availability. *Prevotella intermedia* was identified as a predictor for elevated genetic risk for dementia (*APOE4* carrier status). In contrast, in healthy controls, high concentrations of NO biomarkers were associated with high abundances of *Veillonella–Megasphaera* (ME2) and *Capnocytophaga* (ME7)-dominated microbiome modules, and in healthy controls, no microbiome module correlated with cognitive outcomes. We showed that the genus *Porphyromonas* was more abundant in MCI than in healthy controls, supporting the notion that the presence of elevated oral *Porphyromonas* precedes dementia diagnosis. These results provide a rationale for further research into underlying mechanisms that connect the oral microbiome to cognitive health through the trajectory from health to MCI, and ultimately to postdementia diagnosis. They also highlight the potential for interventions to ameliorate or delay aging-associated cognitive decline through promotion of the *Neisseria–Haemophilus* and eradication of the *Prevotella*-dominated modules of co-occurring oral bacteria.

Within the MCI group, the oral *Neisseria–Haemophilus* module (ME6) was associated with a greater oral nitrite concentration, and a high abundance of the *Prevotella*-dominated module (ME3) was associated with a low oral nitrate concentration. Several *Neisseria* species contain genes that encode proteins for nitrate reduction (*narG*, *napA*) and for the denitrification pathway (*nirK*, *norB*, or *nosZ*; search performed on https://www.uniprot.org/, accessed on 2023 November 20), which restores NO precursors to the circulation thus helping maintain systemic NO availability (34). In contrast, many *Prevotella* species, including *P. intermedia*, *P. jejuni*, and *P. melaninogenica* of the ME3 module in the present study, contain the *nrfA* gene of the dissimilatory pathway of nitrate reduction to ammonia (DNRA; search performed on https://www.uniprot.org/, accessed on 2023 November 20), which “short-circuits” the nitrate–nitrite–NO cycle by removing NO precursors from circulation and decreasing systemic NO availability (34). In healthy controls, the oral nitrite concentration correlated positively with the *Veillonella–Megasphaera* (ME2) module, and oral nitrate with a *Capnocytophaga*-dominated microbiome module (ME7). The latter is consistent with the increase in relative abundance of the *Capnocytophaga* genus following 10 days of high nitrate diet (35), while *Veillonella* species are among the most abundant and potent nitrate-reducing bacteria in the oral cavity (36). In addition to affecting systemic NO homeostasis via the enterosalivary circulation, oral nitrite production represents an important part of host defense: NO and other reactive nitrogen intermediates formed from acidified nitrite inhibit the growth of a wide range of microorganisms (37), and nitrite is bactericidal against oral pathogens including *Fusobacterium nucleatum*, *Eikenella corrodens*, and *P. gingivalis* (38). Diet was not monitored in the present study, and future research is needed to explore how variation in habitual nitrate and macronutrient intakes may influence the relationships between the oral microbiome modules and NO bioavailability. A high oral nitrate:nitrite ratio, characteristic of a nitrate-rich diet (such as the Mediterranean and “Dietary Approaches to Stop Hypertension” diets), favors bacteria of the denitrification pathway (34) which we have shown to be associated with good cognitive outcomes ((35); present study).

A novel finding of the present study was that in the MCI group, the *Neisseria–Haemophilus* module was linked to a broader range of cognitive domains than was seen in the healthy controls. Although module-level correlations with cognitive outcomes did not reach significance in healthy controls, the OTU-level correlation network showed that *Neisseria* genus correlated with working memory. A previous study in healthy older people found a consensus WGCNA correlation between the *Neisseria–Haemophilus* module and a “sustained attention” cognitive domain, which was robust across dietary nitrate and placebo interventions (35). The *Neisseria–Haemophilus* module has been linked to characteristics associated with cognitive health, such as periodontal health, younger age, lower BMI, and nonsmoking status (39). *Neisseria* and *Haemophilus* consistently co-occur in the oral ecosystems (35, 40, 41) and are found in high abundances in saliva (42, 43). Horizontal gene transfer has been shown to occur between *Haemophilus* and *Neisseria meningitidis* (44), suggesting that these genera have a mutually beneficial relationship. Collectively, these results indicate that a high relative abundance of bacteria belonging in the *Neisseria–Haemophilus* module is associated with better cognitive outcomes in individuals with MCI (present study) as well as in healthy older people (35).

There is a significant inflammatory component to the etiology of neurological damage that ultimately manifests as Ad (45). *Prevotella* species play a role as pathobionts or pathogens in inducing periodontitis, a key risk factor for cognitive impairment and Ad (46), and baseline antibodies for *P. intermedia* and *F. nucleatum* have been implicated as predictors of MCI and Ad during a 10-year follow-up (47). The oral microbiome may be one of the factors that initiates the systemic inflammatory cascade ((47); present study), while lifestyle and environmental factors that modulate the oral microbiome may explain some of the individual variation in the progression of neurodegeneration. Within the MCI group, *P. intermedia* inversely correlated with mouth rinse nitrate and nitrite concentrations in the *APOE4* noncarriers, and there was a greater abundance of *P. intermedia* in *APOE4* carriers than in noncarriers. This is reflective of previous findings of higher abundances of Prevotellaceae family members in the gut microbiome of *APOE4* carriers (48). *APOE4* carrier status may therefore be associated with distinct features of both oral and gut microbiomes (48), with important implications for advancing microbiome-targeted preventative therapies. These data also suggest that oral bacteria relative abundances could be used for early detection of risk for cognitive impairment or *APOE4* carrier status.

The *Porphyromonas* genus was identified as a risk predictor for MCI. This is consistent with previous studies showing an association between oral *Porphyromonas* and cognitive decline or Ad symptoms in rats and humans (11, 49–52). *Porphyromonas* spp. are a driver of periodontal disease and have been shown to promote neuroinflammation and degradation through the activation of inflammatory pathways (53). In mice, *P. gingivalis* lipopolysaccharide has been shown to impair learning and memory (54). In the present study, *P. gingivalis* was highly correlated with *F. nucleatum* which has been found to co-occur previously (55). *Fusobacterium nucleatum* is a pathogen that acts as a “scaffolding species” co-aggregating with obligate anaerobes in brain abscesses and in biofilm formation in oral infections (56), and the attachment of *P. gingivalis* to human fibroblasts is enhanced by the presence of *F. nucleatum* (57). *Fusobacterium nucleatum* and *P. gingivalis* are both ubiquitous in the oral cavity and are well-known candidates for causing oral inflammation and periodontal disease (58, 59). Moreover, serum antibodies against *F. nucleatum* and *P. intermedia* are elevated in Ad compared with healthy controls (47). The present findings of a close co-occurrence of *F. nucleatum* and *Po. gingivalis* in the ME4 module, and a significantly elevated *Porphyromonas* in MCI compared with healthy controls, suggest that the co-occurrence of these periodontal pathogens contributes to the development of cognitive impairment.

16S rRNA gene amplicon sequencing has moderate accuracy at species level and, unlike whole genome sequencing, it does not enable verification of nitrate-reductase genes in given OTUs. We relied on a data repository of previously reported bacterial nitrate-reductase genes (https://www.uniprot.org/) in interpreting the present data. Future research should assess the consistency of species-level relationships using metagenomics to improve species-level identification accuracy. It is also important to note that, in addition to the relative abundances of oral bacteria assessed in the present study, total bacterial mass may influence relationships between the oral microbiome and host cognitive function. Nevertheless, the novel findings presented herein based on relative abundance data highlight potential targets for pro- and prebiotic interventions and represent a significant advance in understanding how the oral bacterial ecosystem influences cognition in health and in MCI.

The study participants were comprised mostly of females, which could impact the generalizability of the present results. In females, estrogen may confer protection against stroke and cardiovascular disease by enhancing NO production through increasing endothelial NOS expression and activation (60–62). Sex differences have also been reported in the oral microbiome (63). One study found that after dietary nitrate supplementation, the increase in plasma nitrite concentration and the increase in the extent of the chemical reduction of oral nitrate were both greater in females compared with males, but there were no differences in oral microbiome composition (64). Despite evidence that female sex hormones may confer protection against vascular diseases, women have a higher lifetime risk of developing Ad with potentially fewer modifiable risk factors (65, 66). More research is required to understand the impact of sex hormones on NO bioavailability and the oral microbiome, and to elucidate the potential consequences for the development of MCI and Ad.

The clinical relevance of the present findings is significant, presenting a strong basis for applied research to test the efficacy of nitrate and potentially other nutrients as therapeutic interventions to alter the oral microbiome in MCI and following dementia diagnosis, as well as in *APOE4* carriers and noncarriers. We have shown that dietary nitrate supplementation is a powerful intervention in healthy older people to decrease *P. intermedia* and other pathogenic oral bacteria, including *Clostridium difficile*, *P. gingivalis*, *T. denticola*, and *Tannerella forsythia*, and increase *Neisseria* species (35). There is a need to assess the efficacy of dietary nitrate in individuals at the early stages of cognitive decline, where increased dietary nitrate intake may help reverse the rise in oral *Prevotella:Neisseria* ratio and thus delay the onset of MCI and Ad. Such an intervention may be particularly important for *APOE4* carriers with elevated oral *P. intermedia* preceding dementia diagnosis.

In conclusion, we identified a co-occurring module of oral bacteria dominated by *Neisseria* and *Haemophilus* and associated with oral nitrate reduction via the denitrification pathway, as a positive influence on cognitive outcomes in individuals with MCI. A *Prevotella*-dominated module favoring the DNRA nitrate reduction pathway was associated with low oral NO availability, and *P. intermedia* was revealed as a potential predictor for elevated genetic risk for dementia as indicated by *APOE4* status. These results give rise to a novel hypothesis that the balance between two metabolic pathways for nitrate reduction within the oral ecosystem, denitrification and DNRA, is skewed toward DNRA in MCI and potentially modulated by *APOE4* status.

## Materials and methods

### Participants

All participants were recruited from the PROTECT study, which is an online aging cohort established for tracking the cognitive health of older adults in the United Kingdom (ethics reference number 13/LO/1578, London Bridge National Health Research Ethics Committee). The present study received approval from the University of Exeter Sport and Health Sciences Ethics Committee (190206-B-03), and all participants provided electronic informed consent as part of a validated online registration to take part.

Inclusion criteria for enrollment in PROTECT at the time of this study were ≥50 years of age, no diagnosis of dementia, and access to the internet. This study recruited from the PROTECT cohort for whom genetic data were available in addition to longitudinal cognitive data. Additional eligibility criteria excluded any participants using tobacco, antibiotics, or mouthwash. Participants were randomly selected from healthy and MCI groups in the overall PROTECT cohort, defined by standardized thresholds for cognitive performance as described previously (67). *APOE4* status was identified (*E4E4*, *E3E4*, *E2E4*, *E3E3*, and *E2E3*) and participants were grouped according to genotype.

### Sample collection

The participants were provided with a sample collection kit by post, which included 10 mL of mouthwash (Scope, 15 wt% alcohol, Procter & Gamble) for the self-collection of the oral microbiome and mouth rinse NO biomarker samples. The participants were instructed to collect the mouth rinse sample in the morning and refrain from eating or drinking for at least 2 h prior to sample collection. The participants were asked to swish 10 mL of bactericidal mouthwash (Scope, 15 wt% alcohol, Procter & Gamble) for 30 s. They expectorated the mouth rinse into a universal tube and posted the sample back to the laboratory on the same day as collection. Upon arrival, the samples were immediately stored at −80 °C.

### Oral microbiome 16S rRNA gene amplicon sequencing

The oral bacteria’s genomic DNA was extracted from the mouth rinse samples using a Gentra Puregene Buccal Cell Kit (Qiagen, Germantown, MD, USA), according to the manufacturer’s instructions. The sample libraries were prepared for sequencing using the NEXTflex 16S V1-V3 Amplicon-Seq Kit (Bioo Scientific, Austin, TX, USA) and sequenced using paired-end 300-bp Illumina MiSeq system, as described previously (68). Sequencing data were trimmed using Trim-Galore! (Krueger F. Trim-Galore!, http://www.bioinformatics.babraham.ac.uk/projects/trim_galore/).

Taxonomic classification of the metagenomic sequencing data was performed using the Kraken2 pipeline with the Kraken2 standard build database with a confidence score 0.05 (69).

### NO biomarkers

Mouth rinse nitrate and nitrite concentrations were measured in 1 mL aliquots using ozone-based chemiluminescence, as previously described (70). The mean nitrate and nitrite concentrations from a mouth rinse blank were subtracted from each sample.

### Cognitive tests

Participants performed a series of cognitive tests using a computerized cognitive test system embedded in the PROTECT study website. They were asked to complete the tests three times within a 7-day window, although this was not mandatory. The tests were measures of working memory (Digit Span, Paired Associates Learning, and Self-ordered Search), executive function (Verbal Reasoning, Switching Stroop), and visual attention (Trail Making), and are described in more detail in previous studies (67, 71, 72). The mean of the total scores for each repeat testing session was used for comparison between the two groups, as previously described (67).

### APOE

*APOE* genotyping was performed at deCODE Genetics. DNA was extracted from saliva samples collected by post and genotyping was performed using Illumina Global Screening Array with custom content (including directly genotyped single nucleotide polymorphisms, rs429358 and rs7412, to determine APOE status).

### Statistical analysis

All data processing steps were completed in R statistical software (73). The bacterial OTUs were processed by first removing rare OTUs if there were over 20 missing values across all samples and transformed into relative abundances. There were 148 OTUs used in further analysis. Sample outliers were removed if there were <1,000 bacterial reads present. The remaining samples (healthy, *n* = 60; MCI, *n* = 55) were checked for outliers using hierarchical clustering. NMDS, Shannon H′ diversity index, and Chao1 species richness estimate were completed using the R vegan package (74). NMDS of the oral microbiome in healthy and MCI groups and *APOE* risk groups were based on Bray–Curtis dissimilarity. NMDS ordinations were compared using the ADONIS test in R vegan (74). LEfSe in Conda was used to uncover potential risk predictor OTUs in the samples by comparing the healthy and MCI groups. The samples were normalized, and the alpha value for the factorial Kruskal–Wallis sum-rank test was set to 0.05. The LDA threshold was set to 2.0 and the analysis was completed using the all-against-all “more-strict” method (75).

WGCNA was used to group positively correlated OTUs into modules for analysis against clinical data in a signed network (76). The scale-free topology was calculated for the OTUs. Soft-thresholding power did not reach 0.8, but the data patterns are similar to what has been shown previously (35). Therefore, an arbitrary soft-threshold power was selected according to the recommendations of Langfelder and Horvath (76). Signed correlation networks were used to construct an adjacency matrix for each OTU, where negative correlations were considered unconnected. This adjacency matrix was transformed into a Topological Overlap Matrix and the OTUs were clustered using hierarchical clustering to produce modules. After the OTUs had been grouped into modules, each module eigenvalue was correlated against NO biomarkers and cognitive test results. Correlation network visualization was performed in Cytoscape (77). The significance level was set at *α* < 0.05.

Mouth rinse nitrate and nitrite concentrations were not normally distributed. Therefore, mouth rinse nitrate and nitrite concentrations were compared between Healthy and MCI groups using the Mann–Whitney test, and concentration values are given as the median ± IQR.

## Data Availability

Sequencing data have been deposited in the National Center for Biotechnology Information Sequence Read Archive Database and are available at BioProject PRJNA1106018. Code used for the analysis is available at https://github.com/jlhxx.
